# Effects of Tai Chi Chuan on Cognitive Function in Older Adults with Cognitive Impairment: A Systematic and Meta-Analytic Review

**DOI:** 10.1155/2020/6683302

**Published:** 2020-12-28

**Authors:** Zhidong Cai, Wanting Jiang, Jilin Yin, Zhitong Chen, Jing Wang, Xing Wang

**Affiliations:** ^1^School of Physical Education and Training, Shanghai University of Sport, Shanghai 200438, China; ^2^Physical Education Department, Beibu Gulf University, Qinzhou 535011, China; ^3^School of Physical Education and Health, Shanghai Lixin University of Accounting and Finance, Shanghai 201620, China

## Abstract

This systematic and meta-analytic review aimed to investigate the effects of Tai Chi Chuan (TCC) on the cognitive function of the elderly with cognitive impairment and to analyze the moderators of these effects. We searched eight electronic databases for randomized controlled trials on the effects of TCC on cognitive function, published up to June 14, 2020. The PEDro scale was used to evaluate the methodological quality of the included literature. Stata14.0 software was used for meta-analysis, subgroup analysis, and publication bias testing. A total of 19 studies and 1,970 samples were included. The methodological quality of the included literature was fair to good, and there was no publication bias. Overall, the research shows that the effect of TCC on the elderly with cognitive impairment is statistically significant (SMD = 0.31, *p* < 0.0001). Five of the cognitive function subdomains were significant moderators [*Q* (5) = 15.66, *p*=0.008], and the effect size (ES) was the largest for global cognitive function (SMD = 0.41), followed by executive function (SMD = 0.33), memory (SMD = 0.31), and verbal fluency (SMD = 0.27). Regarding the exercise prescription variables, results were significantly moderated by the length of exercise training [*Q* (2) = 6.00, *p*=0.05], with ESs largest for moderate length (SMD = 0.41), followed by short length (SMD = 0.40) and long length (SMD = 0.29). However, the results were not moderated by session time or frequency. TCC can improve multiple cognitive functions of the elderly with cognitive impairment. The intervention effects are moderated by exercise length, but not by exercise session time and frequency.

## 1. Introduction

As the aged population continuously grows, age-related cognitive decline has become a global public health problem; in particular, the numbers of people with mild cognitive impairment (MCI) and dementia are increasing. MCI is an intermediate stage between normal aging and dementia, and people with MCI are at high risk of developing dementia. The average prevalence of MCI is 16% in the old population [1], and the risk of developing dementia in patients with MCI (10–15%) is much higher than that in healthy old people (1-2%) [2]. The 2015 World Alzheimer's Disease Report predicts that the number of people with dementia worldwide will increase from 46 million to 131.3 million by 2050 [3]. So far, there is no effective drug to delay the cognitive decline caused by MCI and dementia. How to delay the further decline of cognitive function in people with MCI and dementia and how to improve their quality of life in later years have become urgent problems to be solved in aging societies.

A growing number of studies have proved that physical exercise can improve cognitive function. Animal experiments have found that exercise can stimulate neuronal regeneration and reduce the deposition of *β*-amyloid protein, thus alleviating the symptoms of Alzheimer's disease in mice [4]. Research using two-photon in vivo imaging technology revealed that exercise can activate the brain-derived neurotrophic factor (BDNF) pathway in the mouse brain, reduce the loss of apical dendritic spines of neurons, and thereby improve the neural network and the learning ability for motor skills and cognitive function [5]. A large number of clinical experimental studies and systematic reviews have confirmed that physical exercise can improve cognitive function, effectively delay cognitive decline [6], and reduce the risk of dementia [7–10]. In December 2017, the American Academy of Neurology recommended physical exercise to MCI patients [11]. In summary, it is increasingly accepted that physical exercise is an effective nonpharmacological treatment to delay the cognitive decline of the elderly.

Research on exercise interventions to improve cognitive function has mostly used, and proven the effects of, aerobic exercise, but the effect of TCC on cognitive function has received little research attention. TCC perfectly integrates traditional philosophy, the theory of traditional Chinese medicine, and the five-element theory; it also combines physical movement with respiration, mind with consciousness, consciousness with the body, and qi with the body. It strives to achieve a high degree of unity of mind, consciousness, strength, qi, and shape, while constantly adjusting the direction, range, power, and speed of movement. The practice requires not only memory but also a variety of higher-level cognitive functions (such as perceptual speed, visual-spatial ability, attention, multitasking, and planning) to maintain postural stability. Accordingly, the process of movement activates the relevant brain areas and stimulates the excitability of brain cells, which is helpful to strengthen the brain, maintain its perceptual functions, and improve the memory of the elderly [12].

In recent years, many researchers have focused on TCC, and a growing body of empirical studies [13–15] and systematic reviews [16–18] has demonstrated that TCC can effectively improve the cognitive function of the elderly, although some studies have found no relationship between TCC and cognitive decline [19]. One previous meta-analysis only analyzed a few studies and included both healthy individuals and individuals with cognitive impairment in the sample range [20]. Other reviews have included nonrandomized controlled trial (RCT) studies [18, 21], thereby introducing a variety of confounding factors that may lead to inaccurate results. Additionally, previous studies analyzing cognitive function [22] have not considered the moderating effects of different aspects of physical exercise, so the dose-response relationship remains unclear. To address these gaps, this study analyzes the effects of TCC intervention on various cognitive domains and how these are moderated by variation in the dose of physical exercise, aiming to provide a theoretical basis for accurate exercise prescription.

## 2. Methods

This study was performed and reported according to Preferred Reporting Items for Systematic Reviews and Meta-Analyses (PRISMA) [23].

### 2.1. Literature Search Strategy

We searched eight electronic databases (PubMed, Embase, The Cochrane Library, WOS, PsycINFO, CNKI, Wanfang, and China Biology Medicine) from inception to June 14, 2020. Two researchers (C.Z.D and J.W.T) independently used the following search terms (among others) for retrieval: Tai Chi Chuan (TCC), Mild Cognitive Impairment, Alzheimer's disease, dementia, cognitive performance, cognitive function, cognitive processes, executive function, memory, attention, inhibition, shifting, working memory, and randomized controlled trial (RCT). The retrieval strategy adopted the combination of subject words and free words and was determined after repeated prechecking. It was supplemented by manual retrieval of the gray literature and the tracing of previous systematic reviews and references where necessary. Language and publication types were not limited to literature retrieval.

### 2.2. Eligibility Criteria

Two researchers (C.Z.D and J.W.T) independently screened the literature according to the inclusion and exclusion criteria. After the screening, any discrepancy between the two researchers was resolved through consulting the other two researchers (W.N and Y.J.L) until consensus was reached.

The inclusion criteria were as follows: (1) the subjects were elderly with MCI or dementia; (2) the intervention was TCC; (3) all or some of the outcome indicators were cognitive functions; and (4) the study was an RCT.

We set the following exclusion criteria: (1) the subjects were elderly with normal cognition or mental disorders; (2) the intervention program contains confounding factors other than exercise, such as cognitive training, vitamin supplements, and drugs; (3) literature data cannot be extracted, even after contacting the authors; and (4) publications that are qualitative studies, case studies, reviews, nonintervention studies, or conference papers.

### 2.3. Data Extraction

Two researchers (C.Z.D and J.W.T) independently extracted the relevant information using a standardized form. Where data were missing or could not be extracted due to insufficient statistical reporting, we contacted the author(s) to request the missing data.

Extraction contents and coding were as follows. First, we captured the basic details of the literature, including the names and nationalities of authors and the year of publication. Second, we collated and processed the basic details of the subjects, including cognitive status, sample size, age, and education level. Third, we captured data on three exercise prescription variables: frequency, session time, and length [6]. Exercise frequency was classified by the number of exercise sessions per week: low frequency: ≤2 times; moderate frequency: 3-4 times; and high frequency: ≥5 times. Exercise session time was classified as follows: short: ≤45 minutes; moderate: >45 minutes to ≤60 minutes; and long: >60 minutes. Exercise length was classified according to the length of the intervention period: short: 4–12 weeks; midlength: 13–24 weeks; and long: >24 weeks. We did not consider intensity as a moderator because there was a lack of consistent intensity measurement criteria in the included literature. Finally, regarding outcome indicators, all behavioral indicators reflecting cognitive functions were extracted in the form of mean and standard deviation.

### 2.4. Assessment of Study Quality

Methodological quality was evaluated by two researchers (C.Z.D and J.W.T) independently using the Physiotherapy Evidence Database (PEDro) scale [24]. The PEDro scale comprises 11 items: eligibility criteria, randomization, concealed allocation, similar baseline, blinding of subjects, blinding of therapists, blinding of assessors, more than 85% retention, intent-to-treat analysis, between-group comparison, point measure, and measures of variability. The “eligibility criteria” are not scored. One point is scored for each item on which relevant information is explicitly presented, and the maximum score for a study is 10 (9-10 = excellent, 6–8 = good, 4-5 = fair, and <4 = poor).

### 2.5. Statistical Analysis

Stata14.0 software was utilized for data analysis. Extracted data included the mean (M) and standard deviation (SD) of each group at postintervention and the sample size. The standardized mean difference (SMD) was selected as the magnitude of effect sizes (ESs). ESs were calculated by Cohen's *d*, taking 0.2, 0.5, and 0.8 as the respective thresholds for small, medium, and large effects [25]. Heterogeneity was calculated by Higgins's *I*^2^ statistics, taking 75%, 50%, and 25% as the respective thresholds for high, medium, and low ratios of interstudy heterogeneity [26]. Publication bias was tested using the Egger test in Stata14.0.

After calculating the overall ES for cognitive function, subgroup analyzes were conducted for the cognitive function domains (global cognitive function, memory, executive function, attention, verbal fluency, and visual-spatial function), exercise prescription variables (frequency, session time, and length), and cognitive status.

## 3. Results

### 3.1. Literature Search

**Figure 1:**
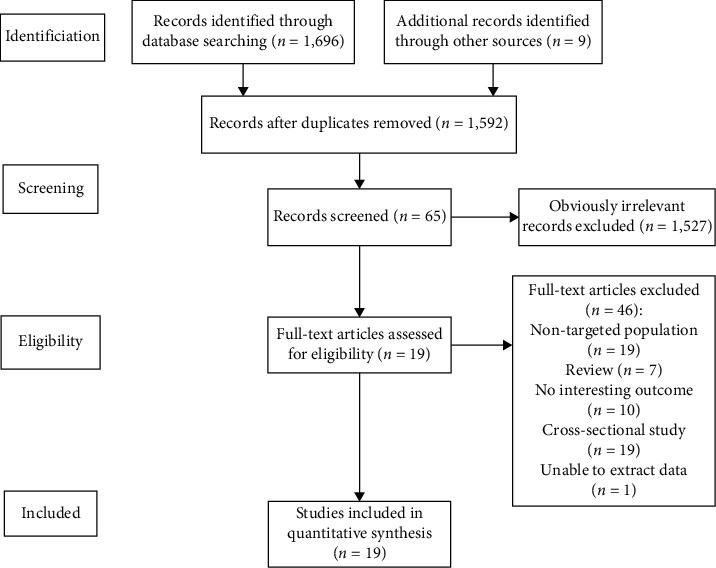
Literature selection flow diagram.

Figure 1 summarizes the flow of the literature search and study selection. The initial search returned 1,705 articles. After removing 113 duplicate articles and 1,527 articles according to the inclusion/exclusion criteria and abstract screening, 19 articles were finally included in the review.

### 3.2. Study Characteristics

Table 1 presents the characteristics of all 19 studies included in this review. The sample size ranged from 36 to 398. The overall sample size was 1,970, including 871 in the experimental groups and 1,099 in the control groups. Among the 19 studies included, 16 focused on MCI elderly and three focused on the elderly with dementia[28, 29, 31]. Participants ranged in age from 66 to 82 years. There were no gender restrictions in any studies, although most participants were women. The included studies were conducted in five countries: eleven in China (57.9%), four in the United States (21.1%), two in Thailand (10.5%), one in Vietnam, and one in France (5.3%).

Sixteen of the 19 studies used Yang-style TCC; of the others, one used Sun-style TCC [44], one used the Westernized version [35], and one did not report this information [36]. Exercise frequency varied from one to five times/week, with three times being most common; exercise session time varied from 20 minutes to 120 minutes, with 60 minutes being most common; and exercise program length varied from 10 weeks to 52 weeks, with 24 weeks being most common. Among the 19 studies, TCC was compared with health education by seven studies [19, 27, 28, 35, 39, 40, 45], no intervention by five studies [31, 37, 41, 43, 46], stretching exercises by three studies [32–34], social activities by three studies [29, 36, 38], and physical training by one study [44].

The main outcomes were six cognitive function areas: global cognitive function, memory, executive function, attention, verbal fluency, and visual-spatial function. The specific test indicators are shown in Table 1. The postintervention mean and standard deviation were compared between the experimental group and the control group. In addition to neurocognitive tasks, some studies explored the cognitive mechanism of TCC through biological and electrophysiological indicators, such as the level of plasma BDNF and magnetic resonance imaging (MRI).

### 3.3. Methodological Quality

The methodological quality of the included studies is reported in Table 2. The PEDro scores of the included studies range from 4 to 10 points, with an average of 6.9 points. The overall methodological quality is fair to good, with PEDro scores ≥6 for 11 studies (good) and PEDro scores of 4-5 for three studies (fair). All the included studies carried out randomization, between-group comparison, point measure, and measures of variability, and 11 achieved more than 85% retention. Eight studies used concealed allocation, blinding of assessors, and intent-to-treat analysis, while three studies used blinding of subjects and blindness of therapists.

### 3.4. Meta-Analysis

**Figure 2:**
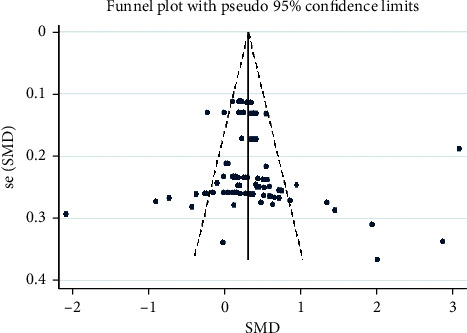
Funnel plot of exercise on inhibition.

A total of 89 effects are included in the meta-analysis, and the overall ES is 0.31, 95% CI (0.28, 0.35), *p* < 0.001, with a statistically significant difference between the experiment and control groups. This indicates that TCC intervention can significantly improve the cognitive function of patients with cognitive impairment. The results of the heterogeneity test revealed a high degree of heterogeneity in the included studies (Table 3), so a random effect model was used to synthesize the data. The funnel plot in Figure 2 is basically symmetrical, which indicates that there is no publication bias. Egger's test shows that there is no publication bias in this study, indicating that the small sample size study does not affect the results (*t* = 1.15, *p* > |*t*| = 0.252 > 0.05) (Table 4) [47, 48].

### 3.5. Subgroup Analysis

#### 3.5.1. Cognitive Function Domain

Subgroup analysis revealed that five subdomains of cognitive function significantly moderated the effect of TCC on cognitive function [*Q* (5) = 15.66, *p*=0.008]. Only visual-spatial function was not a significant moderator. The ES of global cognitive function (Cohen's *d* = 0.41) was greater than that of memory function (Cohen's *d* = 0.31), executive function (Cohen's *d* = 0.33), attention (Cohen's *d* = 0.25), and verbal fluency (Cohen's *d* = 0.27).

#### 3.5.2. Exercise Prescription Variables

The length of the exercise intervention significantly moderated the effect of TCC on cognitive function [*Q* (2) = 6.00, *p*=0.05]. The results of the subgroup analysis indicated that the ES for older adults engaged in a moderate-length TCC intervention (12–24 weeks) (Cohen's *d* *=* 0.41) was larger than that for TCC interventions that were short (<12 weeks) (Cohen's *d* *=* 0.40) or long (>24 weeks) (Cohen's *d* = 0.29).

There were no significant differences among the ESs based upon exercise frequency [*Q* (2) = 1.08, *p*=0.58] or exercise session time [*Q* (2) = 0.21, *p*=0.42].

#### 3.5.3. Cognitive Status

There were no significant differences among the ESs based upon cognitive status [*Q* (1) = 0.04, *p*=0.40].

## 4. Discussion

### 4.1. Overall Analysis of TCC Intervention Effects

To the best of our knowledge, this is the first meta-analysis of RCTs investigating the effects of TCC exercise prescription on cognitive function. It is very important to further understanding of how the exercise prescription potentially moderates the intervention effect. Additionally, no previous meta-analysis has investigated whether cognitive status influences the effect of TCC on cognitive function in the elderly with cognitive impairment.

This meta-analysis included 19 studies and synthesized 89 ESs. The results demonstrate that TCC improves the cognitive function of the elderly with cognitive impairment, with a positive, statistically significant, yet small ES. Based on the results of this review, we believe that TCC is an effective way to improve the cognitive function of elderly with cognitive impairment, which is generally consistent with the results of previous meta-analyses [16, 18, 22]. Techniques such as functional MRI (fMRI) and event-related potential provide further evidence that TCC improves cognitive function. The reviewed studies also provide evidence that TCC improves cognitive function by changing the brain structure [49] and enhancing brain functional connectivity [41, 43], neural activity, and brain electrical activity [50].

### 4.2. Subgroup Analysis of TCC Intervention Effects

#### 4.2.1. Cognitive Function Domains

Although previous studies have shown that TCC has a positive effect on the cognitive function of the elderly, this meta-analysis provides an important extension to the literature by exploring TCC's effects on subdomains of cognitive function. The results of subgroup analysis indicate that TCC has different effects on different cognitive function domains of elderly with cognitive impairment. The results indicate that TCC improves five specific domains of cognitive function: global cognitive function, memory, executive function, attention, and verbal fluency. Conflicting with previous studies, there was no significant effect on visual-spatial function [22]. A cross-sectional study found that open movement can significantly improve visual-spatial ability [51]. The insignificant effect on visual-spatial function in this meta-analysis may be explained by the small number of ESs included (only six), which may not reveal the real effect of the intervention. Although some outcome measures, such as Rey Figure, Clock Drawing, and Block Design, do not show significant improvement, the performance trend in experimental groups was better than in control groups. The findings overall suggest that TCC influences multiple aspects of cognitive function.

Twelve studies analyzed global cognitive function, and they include 23 ESs: 20 positive and three negative. The results of the meta-analysis revealed a significant effect on global cognitive function (Cohen's *d* = 0.40), similar to the findings of previous studies [22]. The main tools used for measuring cognitive function were MMSE, MoCA, ADAS-Cog, CDRS, and MDRS, which are commonly used in cognitive testing. MMSE is the most commonly used cognitive impairment screening scale in clinical practice. It is effective in screening between normal elderly and those with dementia, but not between normal elderly and those with MCI. MoCA has higher sensitivity in the diagnosis of MCI, whereas ADAS-Cog, CDRS, and MDRS have better sensitivity to dementia [52]. In addition to neuropsychological testing tools, Mortimer et al. also used fMRI and reported that 40 weeks of TCC exercise increased brain capacity, thereby improving cognitive function [36]. Sungkarat et al. argued that TCC exercise can significantly improve the executive function and memory of elderly with MCI by upregulating the level of serum BDNF [40]. On closer inspection of the three negative effects reported in the literature, we found that the subjects of Deschamps et al.'s study [19] were frail elderly under long-term nursing, and the control group participated in cognitive action exercises, such as strength training of the upper and lower limbs and breathing exercises. The MMSE scores increased postintervention for both the experimental group and control group, indicating that both TCC and cognitive action exercise can improve cognitive performance. The main purpose of Lavretsky's study was to relieve depression, while the main purpose of Chan et al.'s study [28] was to improve sleep. Although neither study reported significant results, they both showed a trend of improvement. Different research purposes and different disease groups may interfere with the actual effects of TCC intervention.

Memory is an important domain of cognitive function. The results of this study showed that the ES on memory was 0.31. Thirteen included studies analyzed the effect of TCC on memory function, and all 22 ESs showed a positive impact. Although Huang et al. [31] found no difference in immediate recall between the experimental group and control group after 5 months of intervention, there was a significant difference after 10 months of intervention. The intervention effect of TCC on memory function is generally considered substantive. Pesce and Audiffren believes that, for physical exercise interventions on cognitive function, there should be focus not only on the quantitative aspects of exercise prescription (e.g., duration and intensity) but also on the cognitive needs of tasks [53]. TCC contains a rich action structure and relatively fixed sequence, so it requires many cognitive resources to maintain attention, memory, judgment, and decision-making. Among the elderly, these motor learning and coordinated movements may lead to an increase in the volume of gray matter in the middle temporal region, including the hippocampus and frontal parietal lobe network, which may improve memory function [54].

There is a phenomenon of cross overlap in the measures of executive function, attention, and visual-spatial function. Executive function is a high-level cognitive function, including shifting, updating, and inhibition [55]. There are currently 29 methods for testing executive function, each of which focuses on different subcomponents of executive function: examples include the Wisconsin test, Trail Making Test, and Stroop test [56]. A test can also reflect different cognitive areas: for instance, TMT can be regarded as a test of both attention and executive function; CDT can test not only executive function but also visual-spatial function; and DST can test attention, short-term memory, and working memory. Therefore, the differences in the division of cognitive domains lead to differences in the results of meta-analyses. Wayne et al. combined TMT, DST, Stroop, and other indicators and found a large ES (SMD = 0.9) of TCC intervention on executive function [18]; Yang combined attention and processing speed and found a medium ES (SMD = 0.51) and combined abstraction, neural flexibility, and reasoning combine effect and found a small ES (SMD = 0.29). In sum, there is a significant improvement in executive function, attention, and visual-spatial function, but different classifications across meta-analyses lead to substantial differences in ESs.

For verbal fluency, only five ESs (Cohen's *d* = 0.27) were included in this meta-analysis, so the effect of TCC intervention on this domain needs to be confirmed by more studies.

#### 4.2.2. Exercise Prescription Variables

The current meta-analytic takes an important step by evaluating the effects of another group of moderators (exercise prescription) on the effects of TCC on cognitive function. The findings indicate that only exercise length moderates the influence on cognitive function. Among the 19 studies reviewed, four used 4–12-week programs (21.1%), eight used 13–24-week programs (42.1%), and six used programs of more than 24 weeks (31.6%). Most of the included studies adopted the length of an exercise program as 12 weeks or 24 weeks.

This study revealed an inverted U-shaped relationship between cognitive function and exercise program length. The ES of exercise length was the highest for moderate length, followed by short length and finally long length. This result may be due to the “ceiling effect” of TCC on the cognitive function of the elderly with cognitive impairment, such that the effect gradually weakens after 24 weeks, or it may be due to the cognitive decline of patients. In addition, as exercise length increases, the compliance rate may decrease, which may also influence the effect. Previous studies have not reached consensus on the most effective exercise length. Some studies contend that the effect of long programs is greater [57], others argue that short programs are more effective [58], and still others assert that exercise length does not influence the intervention's effectiveness [59]. We suggest that future research should extend the exercise length, increase the number of follow-ups, evaluate whether the cognitive difference between the intervention and control groups increases with age, and encourage the elderly to maintain their exercise habits to improve the compliance rate.

This study's subgroup analysis indicated that exercise session time was not a moderator. This implies that TCC interventions of any exercise session time positively affect cognitive function in older adults with cognitive impairment. However, there were only 3 ESs for 60-minute sessions, so this tentative conclusion should be treated with caution. Many previous studies have revealed that 20 minutes' exercise can significantly improve the cognitive function of the elderly [60, 61]. Very short exercise sessions cannot trigger changes in brain arousal and structure and in body functions, whereas overly long exercise may lead to excessive fatigue in the elderly and no obvious change in brain plasticity. Therefore, a specific causal relationship appears more likely for sessions of moderate duration [62].

The subgroup analysis also indicated that exercise frequency was not a moderator, which suggests that TCC interventions of any exercise frequency result in positive effects for cognitive function in older adults with cognitive impairment. This finding is similar to previously reported results: for instance, Northey et al. found that both low-frequency and moderate-frequency exercise can improve the cognitive function of the elderly [6]. However, because our study only included four articles with high-frequency exercise, our conclusion on this relationship may be unreliable and should be treated with caution.

#### 4.2.3. Cognitive Status

Regarding cognitive status, this study's results show that TCC can significantly improve the cognitive function of people with MCI or dementia, but cognitive state does not moderate the intervention effect. This finding is consistent with those of previous studies [63–65]. A large number of studies have shown that physical exercise can significantly improve the cognitive function of the healthy elderly, elderly with MCI, and dementia patients [66–68].

## 5. Strengths and Limitations

This study's primary strength was the inclusion of RCT studies. In previous studies, the inclusion of cross-sectional studies introduced confounding variables that affect the authenticity of the research results. Another strength of this study is that it analyzed exercise prescription as a set of potential moderators. The results provide a theoretical basis for accurate exercise prescription.

Conversely, our meta-analysis had several limitations, which should be considered in the future research. First, the number of included studies is relatively small, albeit much larger than those of previous meta-analyses. Research on how TCC can influence cognitive function is a relatively recent development, and few experimental studies have been conducted so far, so more research is needed to confirm the nature of the relationship. Second, the included studies have methodological defects, especially the absence of blinding. Third, we only searched Chinese and English databases. Fourth, although most studies use Yang's simplified 24-style TCC, many do not describe the prescription, teaching content, and time allocation, which increases the difficulty of judging the dose-response relationship. It should be noted that TCC is a form of body-mind exercise, which emphasizes not only physical exercise but also psychological training, so the teaching idea, level, and methods of coaches may influence the effect of intervention. Finally, the positive effect of TCC on the cognitive function of the elderly may be moderated by subjects' cardiorespiratory fitness. However, there are not enough data in the included studies to analyze this variable.

## 6. Conclusions

The results of this systematic and meta-analytic review demonstrate that TCC is a promising way to improve the global cognitive function, memory, executive function, attention, and verbal fluency of the elderly with cognitive impairment. Additionally, we observed that exercise length moderated the intervention's influence. However, as neither exercise frequency nor session time appeared to have a moderating effect, the optimal TCC prescription remains unclear.

## Figures and Tables

**Table 1:** Basic characteristics of the included literature.

Author, year	Country	Sample characteristics	Mean age	Cognitive status	Outcome measures	Exercise prescription variables (*F* × *T* × *L*)
Bao, 2019 [27]	China	TCC (*n* = 31)/health education (*n* = 31) Male (*n* = 29)/female (*n* = 33)	67	MCI	Global cognition: MMSE, MoCA Memory: MIC	Yang style 3 × 60 × 24
Chan et al., 2016 [28]	China	TCC (*n* = 27)/health talk (*n* = 25) Male (*n* = 8)/female (*n* = 44)	80	DE	Global cognition: MMSE Memory: MIC	Yang style 2 × 60 × 12
Cheng et al., 2014 [29]	China	TCC (*n* = 35)/handicraft (*n* = 39) Gender unreported	80	DE	Global cognition: MMSE Memory: DSF	Yang style 3 × 60 × 12
Deschamps et al., 2009 [19]	France	TCC (*n* = 15)/health education (*n* = 21) Male (*n* = 22)/female (*n* = 30) (baseline)	81	DE	Global cognition: MMSE	Yang style 4 × 30 × 24
Fogart et al., 2016 [30]	Britain	TCC (*n* = 19)/memory training (*n* = 21) Male (*n* = 19)/female (*n* = 21) (baseline)	72	MCI	Memory: HVLT Executive function: TMT-B	2 × 90 × 10
Huang et al., 2019 [31]	China	TCC (*n* = 36)/routine treatments (*n* = 38) Male (*n* = 24)/female (*n* = 50)	82	MCI	Global cognition: MoCA, MMSE Memory: IR, DR	Yang style 3 × 20 × 40
Lam et al., 2011 [32]	China	TCC (*n* = 171)/stretching (*n* = 218) Male (*n* = 97)/female (*n* = 297)	78	MCI	Global cognition: MMSE, DRC, ADAS-Cog Memory: DR, DSF, MIC Executive function: DSB, CTB Attention: VS, CTA Language: VF	Yang style 3 × 30 × 52
Lam et al., 2012 [33]	China	TCC (*n* = 92)/stretching (*n* = 169) Male (*n* = 92)/female (*n* = 297) (baseline)	78	MCI	Global cognition: MMSE, DRC, ADAS-Cog Memory: DR, DSF, MIC Executive function: DSB, CTB Attention: VS, CTA Language: VF	Yang style 3 × 30 × 52
Lam et al., 2014 [34]	China	TCC (*n* = 96)/ stretching (*n* = 169) Male (*n* = 92)/female (*n* = 297) (baseline)	78	MCI	Global cognition: MMSE, DRC, ADAS-Cog Memory: DR, DSF, MIC Executive function: DSB, CTB Attention: VS, CTA Language: VF	Yang style 3 × 30 × 52
Lavretsky et al., 2011 [35]	USA	TCC (*n* = 33)/health education (*n* = 35) Male (*n* = 28)/female (*n* = 45) (baseline)	71	MCI	Global cognition: MMSE Memory: CVLT Attention: TMT	Yang style 1 × 120 × 10
Mortimer et al., 2012 [36]	USA	TCC (*n* = 30)/no intervention (*n* = 30) Male (*n* = 20)/female (*n* = 40)	68	MCI	Global cognition: MDRS Memory: CAVLT Executive function: SCWT, DSB, TMT-B Language: BNT Visuospatial ability: CDT	Westernized version 3 × 50 × 40
Nguyen and Kruse, 2012 [37]	Vietnam	TCC (*n* = 39)/routine daily activities (*n* = 34) Male (*n* = 48)/female (*n* = 48) (baseline)	69	MCI	Executive function: TMT-B Attention: TMT-A	Yang style 2 × 60 × 24
Sun et al., 2015 [38]	China	TCC (*n* = 72)/playing cards or singing (*n* = 66) Male (*n* = 34)/female (*n* = 104)	69	MCI	Global cognition: MMSE Attention: FAB	Yang style 2 × 60 × 24
Sungkarat et al., 2017 [39]	Thailand	TCC (*n* = 33)/health education (*n* = 33) Male (*n* = 9)/female (*n* = 57)	66	MCI	Memory: DR Executive function: DSB Visuospatial ability: BDT	Yang style 3 × 50 × 15
Sungkarat et al., 2018 [40]	Thailand	TCC (*n* = 33)/health education (*n* = 33) Male (*n* = 9)/female (*n* = 57)	66	MCI	Memory: DR Executive function: DSB Visuospatial ability: BDT	Yang style 3 × 50 × 24
Tao et al., 2016 [41]	China	TCC (*n* = 21)/no intervention (*n* = 25) Male (*n* = 14)/female (*n* = 32)	60	MCI	Memory: WMS	Yang style 5 × 60 × 12
Tao et al., 2017 [42]	China	TCC (*n* = 21)/no intervention (*n* = 25)	60	MCI	Memory: WMS	Yang style 5 × 60 × 12
Tao et al., 2017 [43]	China	Male (*n* = 14)/female (*n* = 32)	60	MCI	Memory: WMS	Yang style 5 × 60 × 12
Taylar-Piliae et al., 2010 [44]	USA	TCC (*n* = 37)/health education (*n* = 56) Male (*n* = 28)/female (*n* = 65)	69	MCI	Memory: SDF Executive function: SDB Language: BNT	Yang style 5 × 45 × 24
Tsai et al., 2013 [45]	USA	TCC (*n* = 28)/health education (*n* = 27) Male (*n* = 15)/female (*n* = 40)	55	MCI	Global cognition: MMSE	Sun style 3 × 30–40 × 20

Note: DE, Dementia; MMSE, Mini-Mental-State Examination; MoCA, Montreal Cognitive Assessment Scale; MIC, Memory Inventory for Chinese; ADAS-Cog, Alzheimer's Disease Assessment Scale-Cognitive Subscale; DRC, Chinese Dementia Rating Scale; MDRS, Mattis Dementia Rating Scale; IVR, immediate verbal recall; DVR, delayed verbal recall; DSF, digit span forward; DSB, digit span backward; HVLT, Hopkins Verbal Learning Test; DR, delayed recall; IR, immediate recall; WMS, Wechsler Memory Scale; TMT-B, Trail Making Test B; CTB, Chinese Trail B; VS, visual span; CTA, Chinese Trail A; FAB, Frontal Assessment Battery; VF, verbal fluency; BNT, Boston Naming Test; CDT, Clock-Drawing Test; BDT, Block Design Test; (*F* × *T*× *L*), frequency × time × length; I/C, Intervention/Control.

**Table 2:** Methodological quality assessment for the included studies.

Reference	Item 1	Item 2	Item 3	Item 4	Item 5	Item 6	Item 7	Item 8	Item 9	Item 10	Sum score
Bao [27]	1	0	1	0	0	0	1	1	1	1	6
Chan et al. [28]	1	1	1	0	0	1	1	1	1	1	8
Cheng et al. [29]	1	0	1	0	0	0	1	1	1	1	6
Deschamps et al. [19]	1	0	0	0	0	1	0	0	1	1	4
Huang et al. [31]	1	1	1	1	1	1	1	1	1	1	10
Lam et al. [32]	1	1	1	0	1	1	1	1	1	1	9
Lavretsky et al. [35]	1	1	1	1	0	1	1	0	1	1	8
Mortimer et al. [36]	1	0	1	0	0	0	1	0	1	1	5
Nguyen and Kruse [37]	1	0	1	0	0	0	1	0	1	1	5
Sun et al. [38]	1	1	1	0	0	0	0	1	1	1	6
Sungkarat et al. [39]	1	1	1	0	0	1	1	1	1	1	8
Tao et al. [43]	1	1	1	1	0	1	0	0	1	1	7
Taylar-Piliae et al. [44]	1	0	1	0	0	0	1	1	1	1	6
Tsai et al. [45]	1	1	1	0	1	1	1	0	1	1	8

Note: Item 1, randomization; Item 2, concealed allocation; Item 3, similar baseline; Item4, blinding of subjects; Item 5, blinding of therapists; Item 6, blinding of assessors; Item 7, more than 85% retention; Item 8, intent-to-treat analysis; Item 9, between-group comparison; Item 10, point measure and measures of variability; 1, explicitly described and present in details; 0, absent, inadequately described, or unclear.

**Table 3:** Summary of meta-analysis and subgroup analysis results.

	*Q* (d*f*)	*I* ^2^ (%)	*n* (ES)	ES (95% CI)	*p*
Overall	**572.48 (88)**, *p* < 0.001	**84.6**	**89**	**SMD** **=** **0.31 (0.28, 0.35)**	**<0.001**
Session time (minutes)	**1.82 (2)**, *p*=0.402				
Short (≤45 min)		86.2	38	SMD = 0.30 (0.25, 0.34)	<0.001
Moderate (45–60 min)		84.3	48	SMD = 0.35 (0.28, 0.42)	<0.001
Long (>60 min)		52.6	3	SMD = 0.30 (0.02, 0.58)	0.03
Frequency (week/times)	**1.08 (2)**, *p*=0.583				
Low (1-2 times)		92.7	12	SMD = 0.32 (0.28, 0.36)	<0.001
Moderate (3-4 times)		82.2	75	SMD = 0.26 (0.13, 0.39)	<0.001
High (≥5 times)		88.4	5	SMD = 0.34 (0.16, 0.62)	0.001
Length	**6.00 (2)**, *p*=0.05				
Short (≤12 week)		73.5	12	SMD *=* 0.40 (0.30, 0.50)	<0.001
Moderate (12–24 week)		85.4	21	SMD *=* 0.41 (0.26, 0.55)	<0.001
Long (>24 week)		85.8	56	SMD *=* 0.29 (0.24, 0.33)	<0.001
Cognitive function domains	**15.66 (5)**, *p*=0.008				
Global cognitive function		67.2	23	SMD = 0.41 (0.33, 0.48)	<0.001
Memory function		68.9	22	SMD = 0.31 (0.22, 0.39)	<0.001
Executive function		77.4	18	SMD = 0.33 (0.25, 0.42)	<0.001
Verbal fluency		0	5	SMD = 0.27 (0.13, 0.41)	<0.001
Attention		96	14	SMD = 0.25 (0.17, 0.34)	<0.001
Visual space function		54.7	7	SMD = 0.03 (−0.28, 0.33)	0.7
Cognitive status	**0.04 (1)**, *p*=0.843				
MCI		85.7	80	SMD *=* 0.33 (0.17, 0.50)	<0.001
DE		56.0	9	SMD *=* 0.31 (0.27, 0.35)	<0.001

**Table 4:** Results of Egger's test.

Std_EFF	Coef.	Std. err.	*t*	*p* > |*t*|	95% CI
Slope	0.1606565	0.1419637	1.13	0.261	−0.1215116, 0.4428247
Bias	0.8837072	0.7670769	1.15	0.252	−0.6409412, 2.408356

## Data Availability

The raw data supporting this manuscript are from previously reported studies and datasets, which have been cited. The processed data are available in the supplementary files.

## Supplementary Materials

Supplementary Materials Supplementary description: the raw data supporting this manuscript are from previously reported studies and datasets, which have been cited. The processed data are available in the supplementary files.

## References

[1] Hu C., Yu D., Sun X., Zhang M., Wang L., Qin H. (2017). The prevalence and progression of mild cognitive impairment among clinic and community populations: a systematic review and meta-analysis. *International Psychogeriatrics*.

[2] Petersen R. C., Doody R., Kurz A. (2001). Current concepts in mild cognitive impairment. *Archives of Neurology*.

[3] Prince M., Wimo A., Guerchet M., Ali G.-C., Wu Y.-T., Prina M. (2015). *World Alzheimer Report 2015*.

[4] Choi S. H., Bylykbashi E., Chatila Z. K. (2018). Combined adult neurogenesis and BDNF mimic exercise effects on cognition in an Alzheimer’s mouse model. *Science (New York, N.Y.)*.

[5] Chen K., Zheng Y., Wei J.-A. (2019). Exercise training improves motor skill learning via selective activation of mTOR. *Science Advances*.

[6] Northey J. M., Cherbuin N., Pumpa K. L., Smee D. J., Rattray B. (2017). Exercise interventions for cognitive function in adults older than 50: a systematic review with meta-analysis. *British Journal of Sports Medicine*.

[7] Kramer A. F., Colcombe S. (2018). Fitness effects on the cognitive function of older adults: a meta-analytic study-revisited. *Perspectives on Psychological Science*.

[8] Bangsbo J., Blackwell J., Boraxbekk C.-J. (2019). Copenhagen consensus statement 2019: physical activity and ageing. *British Journal of Sports Medicine*.

[9] Bossers W. J. R., Van Der Woude L. H. V., Boersma F., Hortobágyi T., Scherder E. J. A., van Heuvelen M. J. G. (2015). A 9-week aerobic and strength training program improves cognitive and motor function in patients with dementia: a randomized, controlled trial. *The American Journal of Geriatric Psychiatry*.

[10] Kirk-Sanchez N. J., Mcgough E. L. (2014). Physical exercise and cognitive performance in the elderly: current perspectives. *Clinical Interventions in Aging*.

[11] Peterson B. M., Johnson C., Case K. R. (2018). Feasibility of a combined aerobic and cognitive training intervention on cognitive function in cancer survivors: a pilot investigation. *Pilot and Feasibility Studies*.

[12] Zou L., Loprinzi P. D., Yeung A. S., Zeng N., Huang T. (2019). The beneficial effects of mind-body exercises for people with mild cognitive impairment: a systematic review with meta-analysis. *Archives of Physical Medicine and Rehabilitation*.

[13] Siu M.-Y., Lee D. T. F. (2018). Effects of Tai Chi on cognition and instrumental activities of daily living in community dwelling older people with mild cognitive impairment. *BMC Geriatrics*.

[14] Xie H., Zhang M., Huo C. (2019). Tai Chi Chuan exercise related change in brain function as assessed by functional near-infrared spectroscopy. *Scientific Reports*.

[15] Wang Q.-b., Sheng Y. (2016). Effect of Tai Ji Quan on cognitive function in old adults with mild cognitive impairment. *Chinese Journal of Rehabilitation Theory and Practice*.

[16] Huy-Leng L. K., Alex P., Michelle P. (2019). The effectiveness of Tai Chi for short-term cognitive function improvement in the early stages of dementia in the elderly: a systematic literature review. *Clinical Interventions in Aging*.

[17] Pan Z., Su X., Fang Q. (2018). The effects of tai chi intervention on healthy elderly by means of neuroimaging and EEG: a systematic review. *Frontiers in Aging Neuroscience*.

[18] Wayne P. M., Walsh J. N., Taylor-Piliae R. E. (2014). Effect of Tai Chi on cognitive performance in older adults: systematic review and meta-analysis. *Journal of the American Geriatrics Society*.

[19] Deschamps A., Onifade C., Decamps A., Bourdel-Marchasson I. (2009). Health-related quality of life in frail institutionalized elderly: effects of a cognition-action intervention and Tai Chi. *Journal of Aging and Physical Activity*.

[20] Zheng W., Xiang Y.-Q., Ungvari G. S. (2017). Tai Chi for mild cognitive impairment: a systematic review. *Psychogeriatrics*.

[21] Wu Y., Wang Y., Burgess E. O., Wu J. (2013). The effects of Tai Chi exercise on cognitive function in older adults: a meta-analysis. *Journal of Sport and Health Science*.

[22] Yang J., Zhang L., Tang Q. (2020). Tai Chi is effective in delaying cognitive decline in older adults with mild cognitive impairment: evidence from a systematic review and meta-analysis. *Evidence-Based Complementary and Alternative Medicine*.

[23] Liberati A., Altman D. G., Tetzlaff J. (2009). The PRISMA statement for reporting systematic reviews and meta-analyses of studies that evaluate health care interventions: explanation and elaboration. *Epidemiology Biostatistics & Public Health*.

[24] Verhagen A. P., De Vet H. C. W., De Bie R. A. (1998). The Delphi list: a criteria list for quality assessment of randomized clinical trials for conducting systematic reviews developed by Delphi consensus. *Journal of Clinical Epidemiology*.

[25] Cohen J., Cohen J., Cohen J. W. (1988). Statistical power analysis for the behavioral science. *Technometrics*.

[26] Higgins J. P. T., Thompson S. G., Deeks J. J. (2003). Measuring inconsistency in meta-analyses. *BMJ*.

[27] Bao N. N. (2019). Study on Taijiquan in patients with amnestic mild cognitive dysfunction. *Medical Information*.

[28] Chan A. W., Yu D. S., Choi K., Lee D. T., Sit J. W., Chan H. Y. (2016). Tai Chi Qigong as a means to improve night-time sleep quality among older adults with cognitive impairment: a pilot randomized controlled trial. *Clinical Interventions in Aging*.

[29] Cheng S.-T., Chow P. K., Song Y.-Q. (2014). Mental and physical activities delay cognitive decline in older persons with dementia. *The American Journal of Geriatric Psychiatry*.

[30] Fogarty J. N., Murphy K. J., Mcfarlane B. (2016). Taoist Tai Chi and memory intervention for individuals with mild cognitive impairment. *Journal of Aging and Physical Activity*.

[31] Huang N., Li W., Rong X. (2019). Effects of a modified Tai Chi program on older people with mild dementia: a randomized controlled trial. *Journal of Alzheimer’s Disease*.

[32] Lam L. C. W., Chau R. C. M., Wong B. M. L. (2011). Interim follow-up of a randomized controlled trial comparing Chinese style mind body (Tai Chi) and stretching exercises on cognitive function in subjects at risk of progressive cognitive decline. *International Journal of Geriatric Psychiatry*.

[33] Lam L. C. W., Chau R. C. M., Wong B. M. L. (2012). A 1-year randomized controlled trial comparing mind body exercise (Tai Chi) with stretching and toning exercise on cognitive function in older Chinese adults at risk of cognitive decline. *Journal of the American Medical Directors Association*.

[34] Lam L. C. W., Chan W. M., Kwok T. C. Y. (2014). Effectiveness of Tai Chi in maintenance of cognitive and functional abilities in mild cognitive impairment: a randomised controlled trial. *Hong Kong Medical Journal*.

[35] Lavretsky H., Alstein L. L., Olmstead R. E. (2011). Complementary use of Tai Chi Chih augments escitalopram treatment of geriatric depression: a randomized controlled trial. *The American Journal of Geriatric Psychiatry*.

[36] Mortimer J. A., Ding D., Borenstein A. R. (2012). Changes in brain volume and cognition in a randomized trial of exercise and social interaction in a community-based sample of non-demented Chinese elders. *Journal of Alzheimer’s Disease*.

[37] Nguyen M. H., Kruse A. (2012). A randomized controlled trial of Tai Chi for balance, sleep quality and cognitive performance in elderly Vietnamese. *Clinical Interventions in Aging*.

[38] Sun J., Kanagawa K., Sasaki J., Ooki S., Xu H., Wang L. (2015). Tai Chi improves cognitive and physical function in the elderly: a randomized controlled trial. *Journal of Physical Therapy Science*.

[39] Sungkarat S., Boripuntakul S., Chattipakorn N., Watcharasaksilp K., Lord S. R. (2017). Effects of Tai Chi on cognition and fall risk in older adults with mild cognitive impairment: a randomized controlled trial. *Journal of the American Geriatrics Society*.

[40] Sungkarat S., Boripuntakul S., Kumfu S., Lord S. R., Chattipakorn N. (2018). Tai Chi improves cognition and plasma BDNF in older adults with mild cognitive impairment: a randomized controlled trial. *Neurorehabilitation and Neural Repair*.

[41] Tao J., Liu J., Egorova N. (2016). Increased hippocampus-medial prefrontal cortex resting-state functional connectivity and memory function after tai chi chuan practice in elder adults. *Frontiers in Aging Neuroscience*.

[42] Tao J., Liu J., Liu W. (2017). Tai Chi Chuan and Baduanjin increase grey matter volume in older adults: a brain imaging study. *Journal of Alzheimer’s Disease*.

[43] Tao J., Chen X., Egorova N. (2017). Tai Chi Chuan and Baduanjin practice modulates functional connectivity of the cognitive control network in older adults. *Scientific Reports*.

[44] Taylor-Piliae R. E., Newell K. A., Cherin R., Lee M. J., King A. C., Haskell W. L. (2010). Effects of Tai Chi and western exercise on physical and cognitive functioning in healthy community-dwelling older adults. *Journal of Aging and Physical Activity*.

[45] Tsai P.-F., Chang J. Y., Beck C., Kuo Y.-F., Keefe F. J. (2013). A pilot cluster-randomized trial of a 20-week Tai Chi program in elders with cognitive impairment and osteoarthritic knee: effects on pain and other health outcomes. *Journal of Pain and Symptom Management*.

[46] Tao J., Chen X., Liu J. (2017). Tai Chi Chuan and Baduanjin mind-body training changes resting-state low-frequency fluctuations in the frontal lobe of older adults: a resting-state fMRI study. *Frontiers in Human Neuroscience*.

[47] Egger M., Smith G. D., Schneider M., Minder C. (1997). Bias in meta-analysis detected by a simple, graphical test. *BMJ*.

[48] Chen F.-T., Etnier J. L., Chan K.-H. (2020). Effects of exercise training interventions on executive function in older adults: a systematic review and meta-analysis. *Sports Medicine*.

[49] Wei G.-X., Dong H.-M., Yang Z. (2014). Tai Chi Chuan optimizes the functional organization of the intrinsic human brain architecture in older adults. *Frontiers in Aging Neuroscience*.

[50] Fong D.-Y., Chi L.-K., Li F. (2014). The benefits of endurance exercise and Tai Chi Chuan for the task-switching aspect of executive function in older adults: an ERP study. *Frontiers in Aging Neuroscience*.

[51] Guo W., Wang B. Y., Ren J. (2019). The mechanism of the advantage of open-skill exercisers on visuospatial working memory in older adults. *China Sport Science and Technology*.

[52] (2018). Chinese guidelines for the diagnosis and treatment of dementia and cognitive impairment. 2018 guidelines for the diagnosis and treatment of dementia and cognitive impairment in China (3): cognitive and functional assessment of dementia. *National Medical Journal of China*.

[53] Pesce C., Audiffren M. (2011). Does acute exercise switch off switch costs? A study with younger and older athletes. *Journal of Sport and Exercise Psychology*.

[54] Janina B., Joenna D., Christian G. (2008). Training-induced brain structure changes in the elderly. *Journal of Neuroscience*.

[55] Miyake A., Friedman N. P., Emerson M. J., Witzki A. H., Howerter A., Wager T. D. (2000). The unity and diversity of executive functions and their contributions to complex “frontal lobe” tasks: a latent variable analysis. *Cognitive Psychology*.

[56] Etnier J. L., Chang Y.-K. (2009). The effect of physical activity on executive function: a brief commentary on definitions, measurement issues, and the current state of the literature. *Journal of Sport and Exercise Psychology*.

[57] Ludyga S., Gerber M., Pühse U., Looser V. N., Kamijo K. (2020). Systematic review and meta-analysis investigating moderators of long-term effects of exercise on cognition in healthy individuals. *Nature Human Behaviour*.

[58] Xue Y., Yang Y., Huang T. (2019). Effects of chronic exercise interventions on executive function among children and adolescents: a systematic review with meta-analysis. *British Journal of Sports Medicine*.

[59] Sanders L. M. J., Hortobagyi T., La Bastide-Van Gemert S. (2019). Dose-response relationship between exercise and cognitive function in older adults with and without cognitive impairment: a systematic review and meta-analysis. *PLoS One*.

[60] Chang Y.-K., Chen F.-T., Kuan G. (2019). Effects of acute exercise duration on the inhibition aspect of executive function in late middle-aged adults. *Frontiers in Aging Neuroscience*.

[61] Chen F.-T., Etnier J., Wu C.-H., Cho Y.-M., Hung T.-M., Chang Y.-K. (2018). Dose-response relationship between exercise duration and executive function in older adults. *Journal of Clinical Medicine*.

[62] Li Z., Peng X., Xiang W., Han J., Li K. (2018). The effect of resistance training on cognitive function in the older adults: a systematic review of randomized clinical trials. *Aging Clinical and Experimental Research*.

[63] Gates N., Fiatarone Singh M. A., Sachdev P. S., Valenzuela M. (2013). The effect of exercise training on cognitive function in older adults with mild cognitive impairment: a meta-analysis of randomized controlled trials. *The American Journal of Geriatric Psychiatry*.

[64] Zheng G., Xia R., Zhou W. (2016). Aerobic exercise ameliorates cognitive function in older adults with mild cognitive impairment: a systematic review and meta-analysis of randomised controlled trials. *British Journal of Sports Medicine*.

[65] Xu Q., Zhao J. L., Wang X. (2019). Meta analysis of the effect of aerobic exercise on cognitive function in patients with Alzheimer’s disease. *Chinese Journal of Rehabilitation Medicine*.

[66] Forbes D., Thiessen E. J., Blake C. M., Forbes S. S., Forbes S. (2014). Exercise programs for people with dementia. *Sao Paulo Medical Journal*.

[67] Erickson K. I., Voss M. W., Prakash R. S. (2011). Exercise training increases size of hippocampus and improves memory. *Proceedings of the National Academy of Sciences*.

[68] Groot C., Hooghiemstra A. M., Raijmakers P. G. H. M. (2016). The effect of physical activity on cognitive function in patients with dementia: a meta-analysis of randomized control trials. *Ageing Research Reviews*.

