# Supplementary material for: Effects of Tai Chi Chuan on Cognitive Function in Older Adults with Cognitive Impairment: A Systematic and Meta-Analytic Review
Source: Evid Based Complement Alternat Med. 2020 Dec 28;2020:6683302. doi: 10.1155/2020/6683302 (PMC7781704; doi:10.1155/2020/6683302)
Supplement: Supplementary Materials — Supplementary description: the raw data supporting this manuscript are from previously reported studies and datasets, which have been cited. The processed data are available in the supplementary files. [file 6683302.f1.docx]

**The data availability statement**

The raw data supporting the manuscript "Effects of Tai Chi on Cognitive Function in Older Adults with Cognitive Impairment: A Systematic Review and Meta-analysis" are from previously reported studies and datasets, which have been cited. The processed data are available in the supplementary files.

**Supplementary Table**

| reference | outcome | exp_n | exp_m | exp_sd | cont_n | cont_m | cont_sd | cognitive domain | frequency | session time | length | cognitive status |
| --- | --- | --- | --- | --- | --- | --- | --- | --- | --- | --- | --- | --- |
| Bao2019 | MMSE | 31 | 25.12 | 4.17 | 31 | 17.91 | 5.66 | GC | 3 | 60 | 24 | MCI |
| Bao2019 | MoCA | 31 | 25.64 | 3.19 | 31 | 19.93 | 2.68 | GC | 3 | 60 | 24 | MCI |
| Chan2016 | MMSE | 27 | 0.9 | 3.5 | 25 | 2.3 | 3 | GC | 2 | 60 | 12 | MCI |
| Chan2016 | MIC | 27 | 2.8 | 11.9 | 25 | 0.9 | 16.96 | Mem | 2 | 60 | 12 | MCI |
| Cheng2014 | MMSE-3month | 39 | 19.3 | 3.9 | 35 | 18.3 | 4.1 | GC | 3 | 60 | 12 | DE |
| Cheng2014 | MMSE-5month | 39 | 19.4 | 3.9 | 35 | 17.2 | 4.1 | GC | 3 | 60 | 12 | DE |
| Cheng2014 | MMSE-9month | 39 | 20 | 3.9 | 35 | 16.2 | 4.1 | GC | 3 | 60 | 12 | DE |
| Cheng2014 | DSF-3month | 39 | 5.9 | 1.8 | 35 | 5.7 | 1.3 | Mem | 3 | 60 | 12 | DE |
| Cheng2014 | DSF-5month | 39 | 6 | 1.8 | 35 | 5.3 | 1.3 | Mem | 3 | 60 | 12 | DE |
| Cheng2014 | DSF-9month | 39 | 6.1 | 1.8 | 35 | 5.2 | 1.3 | Mem | 3 | 60 | 12 | DE |
| Deschamps2009 | MMSE | 15 | 21.1 | 6.4 | 21 | 21.2 | 6.4 | GC | 4 | 30 | 24 | DE |
| Huang2019 | IR-5month | 36 | 8.77 | 4.63 | 38 | 8.8 | 3.19 | Mem | 3 | 20 | 40 | MCI |
| Huang2019 | IR-10month | 36 | 8.8 | 4.92 | 38 | 7.45 | 3.86 | Mem | 3 | 20 | 40 | MCI |
| Huang2019 | DR-5month | 36 | 7.88 | 4.21 | 38 | 7.45 | 3.86 | Mem | 3 | 20 | 40 | MCI |
| Huang2019 | DR-10month | 36 | 7.91 | 3.05 | 38 | 7.52 | 3.17 | Mem | 3 | 20 | 40 | MCI |
| Huang2019 | TMT-B-5month | 36 | -42.9 | 14.11 | 38 | -44.6 | 13.62 | EF | 3 | 20 | 40 | MCI |
| Huang2019 | TMT-B-10month | 36 | -42.5 | 15.06 | 38 | -44.5 | 10.78 | EF | 3 | 20 | 40 | MCI |
| Huang2019 | MMSE-5month | 36 | 21.3 | 6.2 | 38 | 20.24 | 5.41 | GC | 3 | 20 | 40 | MCI |
| Huang2019 | MMSE-10month | 36 | 21.17 | 5.47 | 38 | 19.47 | 5.73 | GC | 3 | 20 | 40 | MCI |
| Huang2019 | MoCA-5month | 36 | 13.94 | 5.88 | 38 | 12.37 | 5.52 | GC | 3 | 20 | 40 | MCI |
| Huang2019 | MoCA-10month | 36 | 14.83 | 5.71 | 38 | 12.16 | 4.72 | GC | 3 | 20 | 40 | MCI |
| Lam2011 | MMSE follow up | 135 | 25.8 | 3.1 | 194 | 25.1 | 3.6 | GC | 3 | 30 | 52 | MCI |
| Lam2011 | ADAS-Cog follow up | 135 | -10.7 | 5.5 | 194 | -12.8 | 6.1 | GC | 3 | 30 | 52 | MCI |
| Lam2011 | Category verbal fluency | 135 | 33.8 | 7.9 | 194 | 31.5 | 7.8 | VF | 3 | 30 | 52 | MCI |
| Lam2011 | Delay recall | 135 | 4.6 | 2.7 | 194 | 4.1 | 2.5 | Mem | 3 | 30 | 52 | MCI |
| Lam2011 | Digit span(forward) | 135 | 6.9 | 2.1 | 194 | 6.5 | 1.4 | Mem | 3 | 30 | 52 | MCI |
| Lam2011 | Digit span(backward) | 135 | 2.6 | 1.3 | 194 | 2.2 | 1.2 | EF | 3 | 30 | 52 | MCI |
| Lam2011 | Visual span(forward) | 135 | 3.6 | 0.9 | 194 | 3.5 | 0.9 | AT | 3 | 30 | 52 | MCI |
| Lam2011 | Visual span(backward) | 135 | 2.6 | 0.9 | 194 | 2.4 | 0.9 | AT | 3 | 30 | 52 | MCI |
| Lam2011 | Chinese Trail A | 135 | -29.3 | 20.6 | 194 | -33.6 | 23.7 | AT | 3 | 30 | 52 | MCI |
| Lam2011 | Chinese Trail B | 135 | -111.4 | 62.5 | 194 | -136.4 | 85.8 | EF | 3 | 30 | 52 | MCI |
| Lam2011 | CDrs, Chinese Dementia rating scale; | 135 | -0.8 | 0.9 | 194 | -1.3 | 1.7 | GC | 3 | 30 | 52 | MCI |
| Lam2012 | MMSE | 92 | 25.4 | 3.3 | 169 | 24.2 | 3.4 | GC | 3 | 30 | 52 | MCI |
| Lam2012 | ADAS-Cog | 92 | -10.4 | 4.7 | 169 | -12.7 | 5.8 | GC | 3 | 30 | 52 | MCI |
| Lam2012 | Category verbal fluency | 92 | 34.6 | 7.5 | 169 | 32.6 | 7.9 | VF | 3 | 30 | 52 | MCI |
| Lam2012 | Delay recall | 92 | 4.9 | 2.3 | 169 | 4 | 2.3 | Mem | 3 | 30 | 52 | MCI |
| Lam2012 | Digit span(forward) | 92 | 6.6 | 1.2 | 169 | 6.3 | 1.3 | Mem | 3 | 30 | 52 | MCI |
| Lam2012 | Digit span(backward) | 92 | 2.4 | 1.2 | 169 | 2.4 | 1.1 | EF | 3 | 30 | 52 | MCI |
| Lam2012 | Visual span(forward) | 92 | 6.6 | 1.3 | 169 | 3.5 | 0.8 | AT | 3 | 30 | 52 | MCI |
| Lam2012 | Visual span(backward) | 92 | 2.2 | 1.1 | 169 | 2.4 | 0.8 | AT | 3 | 30 | 52 | MCI |
| Lam2012 | Chinese Trail A | 92 | -23.7 | 20.3 | 169 | -27.5 | 18.5 | AT | 3 | 30 | 52 | MCI |
| Lam2012 | Chinese Trail B | 92 | -102.3 | 51.6 | 169 | -125.2 | 69.5 | EF | 3 | 30 | 52 | MCI |
| Lam2012 | CDrs, Chinese Dementia rating scale; | 92 | -0.89 | 0.97 | 169 | -1.58 | 1.37 | GC | 3 | 30 | 52 | MCI |
| Lavretsky2011 | MMSE | 33 | 29.2 | 1.1 | 35 | 29.3 | 1.1 | GC | 1 | 120 | 10 | MCI |
| Lavretsky2011 | Long delayed recall | 33 | 12.36 | 2.8 | 35 | 10.5 | 3.4 | Mem | 1 | 120 | 10 | MCI |
| Lavretsky2011 | trailA errors | 33 | -0.19 | 0.47 | 35 | -0.54 | 1.09 | AT | 1 | 120 | 10 | MCI |
| Mortimer2012 | digit span forward | 30 | -0.28 | 1.03 | 30 | 0.48 | 1.08 | Mem | 3 | 50 | 40 | MCI |
| Mortimer2012 | DSB | 30 | 0.41 | 1.43 | 30 | 0.22 | 1.28 | EF | 3 | 50 | 40 | MCI |
| Mortimer2012 | REYfigure | 30 | -1.03 | 4.03 | 30 | -1 | 2.45 | VS | 3 | 50 | 40 | MCI |
| Mortimer2012 | rey recall | 30 | -0.31 | 22.81 | 30 | 3.44 | 4.89 | VS | 3 | 50 | 40 | MCI |
| Mortimer2012 | Bell cancellation test | 30 | -0.14 | 1.43 | 30 | 0.3 | 0.93 | VS | 3 | 50 | 40 | MCI |
| Mortimer2012 | stroop word | 30 | -0.17 | 0.38 | 30 | -0.48 | 1.31 | AT | 3 | 50 | 40 | MCI |
| Mortimer2012 | stroop color | 30 | -1.03 | 2.16 | 30 | -1.26 | 1.96 | AT | 3 | 50 | 40 | MCI |
| Mortimer2012 | stroop color-word | 30 | 3.07 | 6.41 | 30 | 2.74 | 5.87 | EF | 3 | 50 | 40 | MCI |
| Mortimer2012 | auditory verval learning test (Irecall) | 30 | 2.86 | 2.22 | 30 | 2.48 | 2.21 | Mem | 3 | 50 | 40 | MCI |
| Mortimer2012 | auditory verval learning test (Drecall) | 30 | 4.48 | 3.17 | 30 | 3.26 | 3.22 | Mem | 3 | 50 | 40 | MCI |
| Mortimer2012 | delayed recognition | 30 | 4.66 | 3.27 | 30 | 2.65 | 3.2 | Mem | 3 | 50 | 40 | MCI |
| Mortimer2012 | cateory verval fluency | 30 | -0.38 | 4.18 | 30 | -2.91 | 4.4 | VF | 3 | 50 | 40 | MCI |
| Mortimer2012 | WAIS Similarities | 30 | 2.43 | 3.91 | 30 | 1.48 | 2.87 | EF | 3 | 50 | 40 | MCI |
| Mortimer2012 | TMTA | 30 | -11.17 | 17.47 | 30 | 4.24 | 16.63 | AT | 3 | 50 | 40 | MCI |
| Mortimer2012 | TMTB | 30 | 38.21 | 65.35 | 30 | -16.9 | 62.4 | EF | 3 | 50 | 40 | MCI |
| Mortimer2012 | Clock drawing | 30 | 1.52 | 4.18 | 30 | 2.14 | 4.36 | VS | 3 | 50 | 40 | MCI |
| Mortimer2012 | boston naming (correct name) | 30 | 1.76 | 2.01 | 30 | 1.17 | 3.38 | VF | 3 | 50 | 40 | MCI |
| Mortimer2012 | Mattis dementing rating scale(total) | 30 | 4.59 | 6.73 | 30 | 0 | 5.98 | GC | 3 | 50 | 40 | MCI |
| Mortimer2012 | Mattis attention | 30 | 0.48 | 0.99 | 30 | -0.04 | 0.71 | AT | 3 | 50 | 40 | MCI |
| Mortimer2012 | Mattis initiation | 30 | 1.06 | 4.03 | 30 | -1.26 | 2.91 | EF | 3 | 50 | 40 | MCI |
| Mortimer2012 | Mattis construction | 30 | -0.14 | 0.58 | 30 | 0.13 | 1.39 | VS | 3 | 50 | 40 | MCI |
| Mortimer2012 | Mattis conceptalization | 30 | 1.52 | 3.25 | 30 | 0.52 | 2.29 | EF | 3 | 50 | 40 | MCI |
| Mortimer2012 | Mattis memory | 30 | 1.66 | 2.14 | 30 | 0.65 | 1.75 | Mem | 3 | 50 | 40 | MCI |
| Nguyen2012 | TMT-A | 39 | 35.05 | 4.31 | 34 | 44.24 | 4.54 | AT | 2 | 60 | 24 | MCI |
| Nguyen2012 | TMT-B | 39 | -102.05 | 5.01 | 34 | -118.32 | 6.36 | EF | 2 | 60 | 40 | MCI |
| Sun2015 | MMSE-3month | 72 | 27.8 | 1.7 | 66 | 27.1 | 1.8 | GC | 2 | 60 | 24 | MCI |
| Sun2015 | MMSE-6month | 72 | 28 | 2 | 66 | 27.3 | 1.9 | GC | 2 | 60 | 24 | MCI |
| Sun2015 | FAB(Frontal Assessment Battery7-18)3月 | 72 | 14.9 | 2.4 | 66 | 14.3 | 2.8 | AT | 2 | 60 | 24 | MCI |
| Sun2015 | FAB(Frontal Assessment Battery7-18）6月 | 72 | 15.7 | 2.4 | 66 | 14.5 | 3.2 | AT | 2 | 60 | 24 | MCI |
| Suntkarat2017 | Logical Memory delayed | 33 | 31.8 | 7.4 | 33 | 25.6 | 8.9 | Mem | 3 | 50 | 15 | MCI |
| Suntkarat2017 | digit span forward/backward | 33 | 13.8 | 3.2 | 33 | 13.2 | 2.6 | EF | 3 | 50 | 15 | MCI |
| Suntkarat2017 | block design | 33 | 20.4 | 5.5 | 33 | 16.9 | 7.6 | VS | 3 | 50 | 15 | MCI |
| Suntkarat2017 | TMT B-A | 33 | -71.4 | 34.2 | 33 | -107.9 | 63.3 | EF | 3 | 50 | 15 | MCI |
| Suntkarat2018 | Logical Memory delayed | 33 | 33.5 | 9.5 | 33 | 21.9 | 7.6 | Mem | 3 | 50 | 15 | MCI |
| Suntkarat2018 | digit span forward/backward | 33 | 12.9 | 2.7 | 33 | 12.5 | 1.8 | EF | 3 | 50 | 15 | MCI |
| Suntkarat2018 | block design | 33 | 19.9 | 6.1 | 33 | 17.1 | 6.9 | VS | 3 | 50 | 15 | MCI |
| Suntkarat2018 | TMT B-A | 33 | -76.2 | 46.7 | 33 | -101.1 | 60.8 | EF | 3 | 50 | 15 | MCI |
| Tao2017 | memory quotient | 21 | 123.57 | 11.42 | 25 | 97.76 | 13.92 | Mem | 5 | 60 | 12 | MCI |
| Taylar2010 | animal-naming | 37 | 21.5 | 4.1 | 56 | 21.3 | 4.1 | VF | 5 | 45 | 24 | MCI |
| Taylar2010 | digits forward | 37 | 9.73 | 1.8 | 56 | 9.65 | 4.1 | Mem | 5 | 45 | 24 | MCI |
| Taylar2010 | digits backward | 37 | 7.1 | 1.7 | 56 | 6.2 | 1.6 | EF | 5 | 45 | 24 | MCI |
| Tsai2013 | MMSE-9week | 28 | 27.33 | 2.33 | 27 | 25.85 | 2.33 | GC | 3 | 35 | 20 | MCI |
| Tsai2013 | MMSE-21week | 28 | 27.04 | 2.91 | 27 | 25.63 | 2.91 | GC | 3 | 35 | 20 | MCI |

Note：exp_n, sample size of experimental group; exp_m, mean value of experimental group; exp_sd, standard deviation of experimental group; cont_n, sample size of control group; cont_m, mean value of control group; cont_sd, standard deviation of control group; MMSE, Mini-Mental State Examination; mini mental; MoCA, Montreal Cognitive Assessment Scale; MIC, Memory inventory for Chinese; ADAS-Cog, Alzheimer’s Disease Assessment Scale-Cognitive subscale; DRC, Chinese Dementia rating scale; MDRS, Mattis Dementia Rating Scale; IVR, immediate verbal recall; DVR, delayed verbal recall; DSF, digit span forward; DSB, digit span backward; HVLT, Hopkins Verbal Learning Test; DR, delayed recall; IR, immediate recall; WMS, Wechsler Memory Scale; TMT-B, Trail Making Test B; CTB, Chinese Trail B; VS, visual span; CTA, Chinese Trail A; FAB, Frontal Assessment Battery; VF, verbal fluency; BNT, Boston Naming Test; CDT, Clock-Drawing Test; BDT, the Block Design Test; GC, global cognitive function; Mem, memory; EF, executive function; VF, verbal fluency; vs, visual span; AT, attention.
